# The time geography of segregation during working hours

**DOI:** 10.1098/rsos.180749

**Published:** 2018-10-03

**Authors:** Teodoro Dannemann, Boris Sotomayor-Gómez, Horacio Samaniego

**Affiliations:** Laboratorio de Ecoinformática, Universidad Austral de Chile, Campus Isla Teja, Valdivia, Chile

**Keywords:** segregation, community detection, network analysis, urban dynamics

## Abstract

While segregation is usually evaluated at the residential level, the recent influx of large streams of data describing urbanites’ movement across the city allows to generate detailed descriptions of spatio-temporal segregation patterns across the activity space of individuals. For instance, segregation across the activity space is usually thought to be lower compared with residential segregation given the importance of social complementarity, among other factors, shaping the economies of cities. However, these new dynamic approaches to segregation convey important methodological challenges. This paper proposes a methodological framework to investigate segregation during working hours. Our approach combines three well-known mathematical tools: community detection algorithms, segregation metrics and random walk analysis. Using Santiago (Chile) as our model system, we build a detailed home–work commuting network from a large dataset of mobile phone pings and spatially partition the city into several communities. We then evaluate the probability that two persons at their work location will come from the same community. Finally, a randomization analysis of commuting distances and angles corroborates the strong segregation description for Santiago provided by the sociological literature. While our findings highlights the benefit of developing new approaches to understand dynamic processes in the urban environment, unveiling counterintuitive patterns such as segregation at our workplace also shows a specific example in which the exposure dimension of segregation is successfully studied using the growingly available streams of highly detailed anonymized mobile phone registries.

## 1. Introduction

The historical and unprecedented growth of income inequality worldwide has pushed segregation to a pivotal concept in the description of social systems [1]. For instance, segregation is known to generate far reaching impacts for individuals and groups by altering opportunities for education, employment, health care and general welfare, among others. Segregation has extensively been described to effect societal imbalances leading to critical states in terms of security, health and wealth distribution [2–5], while social cohesion has been posed as a fundamental process fostering the enrichment of the social capital available at particular locations [6–8]. However, defining and measuring segregation remains a complex and elusive task in which scientists have recognized several dimensions, which are not integrated in a general conceptual framework [9]. So far, standard methods have mostly relied on static views seeking to describe and characterize residential ghettoization [10,11] and several indices have been put forward to quantify inequality across residential areas [12], the most paradigmatic example being the Duncan dissimilarity index [13], which measures the percentage of minority population that would have to be relocated in order to perfectly integrate among the distribution of residents of a region [14]. However, these indices do not often consider social interactions in other contexts such as work and leisure to define segregation. Such vertical view of social integration seems thus to be a fundamental aspect that, while embedded in the concept of segregation, is not often considered for the study of the geography of inequality. The explicit consideration of this latter approach has led to new insights for the study of segregation that mostly focus on the so-called *exposure dimension* of segregation in an attempt to capture ‘the extent in which members of one group encounter members of another group in their local spatial environments’ [15,16]. The concept of exposure explicitly takes into account the set of spaces that every person visits during his daily journey, also called the activity space within the subdiscipline of time geography [17,18]. For instance, Wong & Shaw [11] used daily travel data surveys in conjunction with racial-ethnic data to calculate the exposure (or, conversely, the isolation) level of different ethnic groups in southeast Florida. Similarly, Farber *et al.* [14] used the time-geography framework and origin–destination surveys to estimate the social interaction potential index, given by the spatio-temporal prism generated between all possible paths between home and work.

Nowadays, the explosive use of communication technologies, such as cellphones, have made huge volumes of non-conventional data available for research purposes. For instance, by knowing to which cellphone tower we connect across the day permits the reconstruction of urbanites’ daily trajectories, thereby providing a surprisingly high spatio-temporal resolution of social interactions [19]. This approach has been widely used recently to assess a variety of topics going from individual mobility patterns [20] and land-use patterns [21], to the detection of relevant places of high social activity within the city [22], thus unveiling the dynamic structure of cities [23,24]. Ratti *et al*. [25] used Newman’s community detection algorithm [26] on call detail records (CDR) of landline communication in the UK to unveil zones of common social interactions. Interestingly, these automatically detected communities show high correlation with administrative regions of Great Britain. Since then, several studies of mobility networks have been published unveiling meaningful communities out of social activities at the level of individual cities, regions and countries [27–29]. However, some concern has been voiced given the simplicity of the null model used in Newman’s algorithm (i.e. the Erdős–Rényi network, a purely random network) as detected communities could simply be a consequence of the local movement of individuals. More realistic approaches have since emerged to include gravitational effects within the null model [30,31].

In this contribution, we combine community detection algorithms [32], an index of social segregation (i.e. isolation) [16] and random walk analysis to provide a robust description of urban segregation. We aim to provide a tool-set that will highlight existing levels of social interaction between the members of different communities in their urban context. We hence delineate communities with no external categorization (such as socioeconomic level, or other demographic variable) that naturally emerge by applying Louvain’s community detection method [32] over the network generated from urbanites’ trajectories travelling from home to work. We finally compare our detected communities with zones that have been qualitatively well described by the sociological literature in our model city (i.e. Santiago), finding a high correspondence between them. These findings corroborate the well-known isolation pattern among different socioeconomic groups.

## 2. Material and methods

### 2.1. Dataset

We use anonymized CDR data of mobile phone users at a spatial resolution of individual cellphone towers. Data were provided by Telefónica Chile and represent a 37% share of the mobile phone market in Chile. Conversely to voice CDR, the dataset analysed consists of all cellphone pings (i.e. data) to an antenna along four working weeks (from Monday to Friday) in March, May, October and November 2015, summing a total of 9 × 10^8^ call records representing 3.5 × 10^5^ individual users in Santiago, Chile. These datasets are also known as XDR (e.g. [33]). Only cellphone towers within the urban boundary were considered, based on the official administrative registries [34]. Voronoi tesselations were constructed around each tower to represent its spatial coverage area. Rural cellphone towers were discarded imposing a minimum of 70% overlap between its Voronoi area and the urban area.

### 2.2. Home and workplace definition

Home and work locations were inferred following procedures outlined in Phithakkitnukoon *et al.* [35] and Šćepanović *et al.* [36]. While other procedures have been developed to accurately unveil home locations, e.g. Vanhoof *et al*. [37], the high temporal density of our XDR data allow the use of simple heuristics. We define the home location for each user as the most frequented tower between 22.00 and 07.00. Likewise, work location was defined as the place with more pings between 09.00 and 17.00. We only consider users with at least five pings to both home and work locations that had made over 50% of their total pings at those places during the entire year of analysis. This additionally allows to minimize uncertainties related to seasonal effects (e.g. changing movement behaviour of people going on holidays) among others.

### 2.3. Network construction and community detection

Undirected weighted networks were built based on home–work (H-W) trajectories per user. Nodes represent towers and weighted links the number of H-W trajectories shared by two towers. Louvain’s community detection algorithm [32] was used to spatially segment towers in the network and to identify common properties among H-W travel behaviour of corresponding Voronoi residents. Louvain’s algorithm is based on the maximization of the network modularity by measuring the density of links inside each community as compared to links between communities [26,32,38]. We used this method because it has shown to be of high performance in terms of accuracy and computing time compared to other methods [39]. Even after filtering, some antennas remained without affiliation to any of the detected communities forming interspersed communities of individual antennas. We discarded those nodes and labelled each user with the community corresponding to its home location.

The main network corresponds to the one generated for all four weeks of data. However, we additionally generated a network for each working day of the week, and compared the communities generated in each one with respect to the main (aggregated) network (see electronic supplementary material, S1).

### 2.4. Isolation index as a measure of segregation

As Massey & Denton [16] proposed in their seminal work, isolation of a certain group (e.g. community) C can be measured as

2.1 $$P_{C}=\sum_{i=1}^{n}\frac{c_{i}}{C}\frac{c_{i}}{T_{i}},$$

where, in our case, *c*_*i*_ is the number of cellphone users of community C working in area unit (Voronoi cell) *i*, *C* is the total number of users belonging to community C, and *T*_*i*_ the total count of users working in unit *i*. Each user is assigned to the community detected for its home location. Consequently, *c*_*i*_/*C* denotes the probability that a randomly picked member of community C will work in unit *i*, and *c*_*i*_/*T*_*i*_ is the fraction of users working in unit *i* belonging to community C, the amount a specific community contributes to the total number of workers in unit *i*. Hence, $P_{C}$ is the probability that a user of community C will randomly interact with someone of its same community at its work location *i* (i.e. picking two members of the same community C working within the same Voronoi cell). This makes equation (2.1) a direct measure of the level of isolation among members of your own community (C) while at work. Thus, large $P_{C}$ (approx. 1) is, in fact, an indicator of highly segregated communities, while smaller values will denote more integrated ones. As defined here, $P_{C}$ is bounded between two limit cases: The first case may be thought of as the ‘well-mixed limit’, i.e. if for each user, work location was chosen completely at random and therefore members of all communities mingle. Then, *c*_*i*_}/*T*_*i*_ = *k*, with *k* the proportion of group C to the total population. Replacing this in equation (2.1), we obtain $P_{C}=k$, showing that, when the population is completely mixed, the probability of encounter between members of the same community is merely the proportion of this community to the total population size. In the other limit, the population is completely segregated (i.e. isolated) and cellphone users belonging to a particular community will share their workplace only with members of their own community C (i.e. *c*_*i*_/*T*_*i*_ = 1), and one easily gets $P_{C}\to 1$. We calculated real isolation indices (RII) for each community from our dataset, and a simulated isolation index (SII) from simulations performed as follows.

### 2.5. Random walks and robustness of community detection

Using a randomization procedure, we evaluated the likelihood of obtaining the real H-W trajectories and their respective isolation indices. To such aim, we characterized the displacement of users by recovering the statistical distribution of (i) H-W distances (*D*_HW_) and (ii) angles of direction (*theta*) of each H-W journey with respect to the east direction for each community. We then randomly draw *D*_HW_ and *theta* for each user from the empirical distribution corresponding to its community to obtain a new simulated work location. From this, and by maintaining the original home location (i.e. Voronoi cell), we obtained new simulated H-W trajectories allowing us to compute a simulated isolation index (SII) to compare against RII. Further details on these simulations are included in the electronic supplementary material. Notice that, although directionality is important when constructing our null model, we neglect this when constructing our network and its subsequent communities because our focus is to understand how the city is fragmented, irrespective of the commuting direction of users. Note that by doing this, we are assuming that a person that travels from point *A* to point *B* will connect both points of the city in the same way that a commuter going from point *B* to *A* would do.

### 2.6. Socioeconomic composition of communities

**Figure 1.**
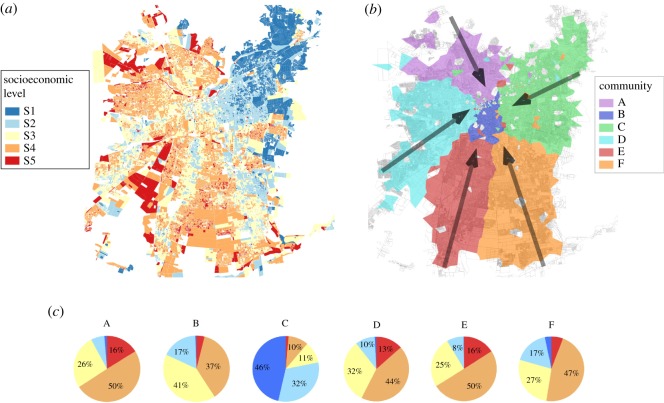
Santiago, Chile. (*a*) Spatial distribution of socioeconomic level. (*b*) Spatial distribution of detected communities using Louvain’s algorithm. Arrows represent the mode of angles in H-W commuting trajectories for each community. The length and angle (*theta*) of arrows are proportional to the distance and direction of H-W commuting, and (*c*) socioeconomic level composition of each detected community.

The classification of socioeconomic level (SEL) is taken from Adimark [40] (figure 1*a*), which defines SEL from the national census data taking into account two dimensions: educational level and the ownership of material assets (see electronic supplementary material, S3, for further methodological details). Adimark identifies five relevant groups labelled: S1, S2, S3, S4 and S5, with S1 as the most affluent group and S5 the group with the lowest income and educational achievements. Spatially, each census block is assigned to one SEL group. Because blocks are variable in size and shape, SEL was assigned to a Voronoi cell by weighing SEL of each census block by the areal contribution to each cell (see electronic supplementary material, S4, for further details). We then aggregated all Voronoi cells corresponding to a specific community to obtain the final SEL composition of each community.

## 3. Results

### 3.1. Description of communities

Six communities were retrieved from Santiago’s H-W aggregated network (figure 1*b*). Notably, daily networks were also split into six communities highly consistent with communities detected in the aggregated network (see electronic supplementary material, S1, with detailed community changes across daily networks). Table 1 shows the percentage of nodes (cellphone towers) for each weekday that retains its community affiliation in the aggregated network. At least 75% of such nodes retain their community affiliation, independent of the weekday chosen.

**Table 1.** Percentage of nodes retaining community affiliation during weekdays as compared to the aggregated network.

	Monday	Tuesday	Wednesday	Thursday	Friday
retained nodes	81.21%	77.53%	80.59%	75.26%	79.28%

A qualitative comparison of figure 1*a* and 1*b* shows an intriguing correspondence between the distribution of SEL and detected communities, respectively. Figure 1*c* complements such view by highlighting the specific SEL composition of each detected community. Community C has, by far, the highest fraction of most affluent SEL (S1 and S2). These groups, however, constitute less than 10% of communities A, D and E, where S3 and S4 dominate. Community F resembles A, D and E, but has a larger presence of more affluent users. Finally, group B is in between extremes, and has a high composition of middle SEL (S3).

**Figure 2.**
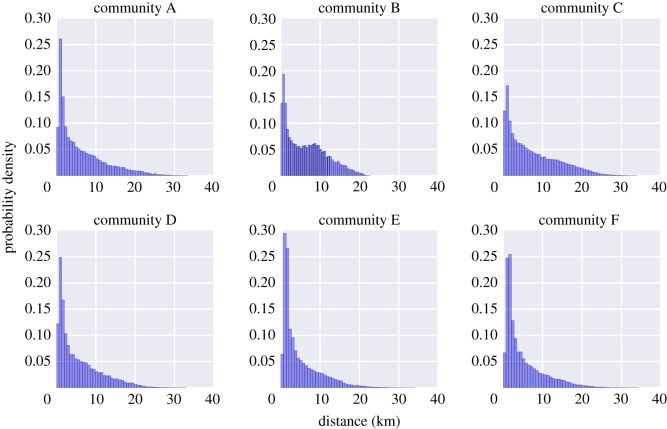
Probability distribution of commuting distance among individuals affiliated to each of the six communities detected in Santiago, Chile.

**Figure 3.**
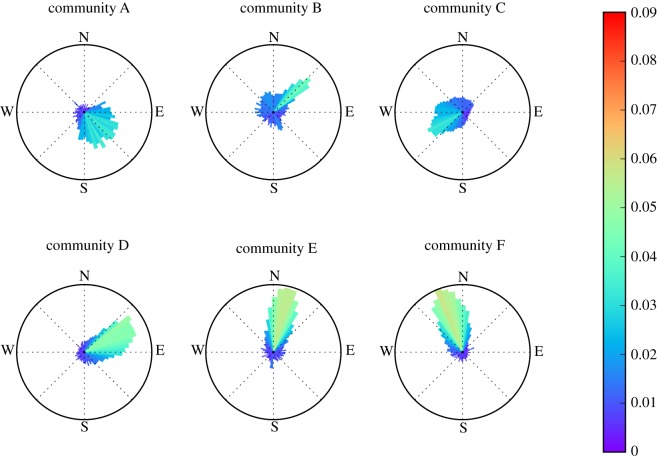
Probability distribution of H-W commuting angles across individuals affiliated to each of the six communities detected in Santiago, Chile. Colour bar shows the probability density estimation in the direction angles (*theta*) of the polar plot.

H-W distances across most communities have a monotonic positive skew (figure 2) with a small mode averaging 1.25 (table 2) in spite of having a mean journey to work of 5–7 km. Community B is the only non-monotonic probability density with a second mode just under the 10 km. Similarly, the distribution of H-W angles (figure 3) suggests a radial movement configuration pointing to the centre of the city (i.e. community B), denoting average commuting direction (*theta*) and distances for each community (see also vector showing *theta* and magnitude in figure 1*b*).

**Table 2.** Mode and standard deviation (s.d.) of commuting distance for each detected community.

	communities					
	A	B	C	D	E	F
mode (km)	1.55	1.01	0.89	1.20	1.30	1.55
s.d. (km)	5.97	5.11	6.37	5.32	4.71	4.93

### 3.2. Isolation and H-W segregation

**Figure 4.**
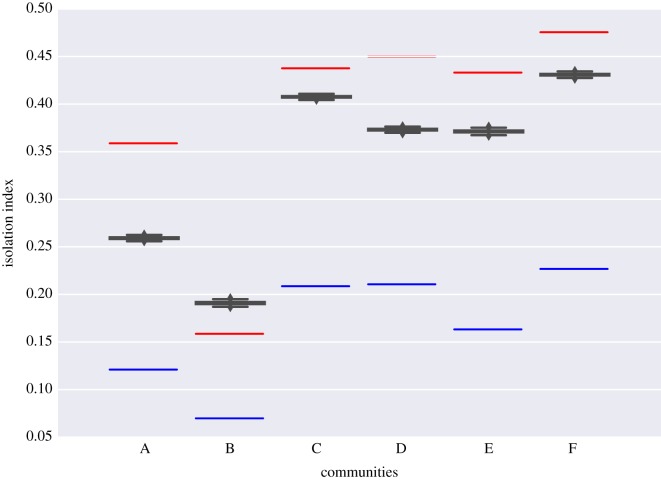
Isolation index value of detected communities. Real isolation index (RII) values are shown as red lines, while values obtained from simulations (SII) are depicted by black boxplots. Blue lines show isolation index values in the ‘well-mixed limit’, as explained in §2.

The comparison of RII to SII, in figure 4, shows that five out of six communities have real values (red segments) much larger than expected based on our simulation framework. Community B seems to be the only exception and shows smaller RII values (0.159) compared with randomized values (SII = 0.191) suggesting this region as the only one that is not statistically segregated (downtown Santiago, mainly). Specific isolation index values are shown in table 3. The distance of RII from SII, in terms of the standard deviation of simulations (*σ*_SII_) shows the likelihood to obtain RII from our simulation. The blue segments in figure 4 show the isolation index value in the hypothetical ‘well-mixed limit’, that is, drawing workplaces not from the known *theta* and *D*_HW_, but from a completely random uniform distribution independent from their home location.

**Table 3.** Comparison of isolation indexes obtained from empirical data (RII, red segment in figure 4) and simulations obtained from randomization (SII, black boxes in figure 4). Average values 〈SII〉 and standard deviations *σ*_SII_ are shown. The last row shows the separation of RII with respect to SII in standard deviation units, *σ*_SII_.

	communities					
	A	B	C	D	E	F
RII	0.359	0.159	0.438	0.450	0.433	0.476
〈SII〉	0.259	0.191	0.408	0.373	0.371	0.431
*σ*_SII_	0.0013	0.0016	0.0011	0.0012	0.0015	0.0013
$\frac{|\mathrm{RII}-\langle \mathrm{SII}\rangle |}{\sigma_{\mathrm{SII}}}$	74.7	20.08	26.19	64.09	42.04	35.28

## 4. Discussion

Understanding segregation has proven to be a multidimensional issue highly regarded by sociologists, economists and social planners in general. We here propose a methodological framework that delves into the exposure dimension of segregation among urbanites while they are at their work location. We employ the Louvain method [32] to outline communities representing isolated social ‘bubbles’, or groups, in terms of their commuting behaviour, and use an isolation index to evaluate the degree of interaction between individuals of these groups during working hours.

The novelty of combining community detection algorithms with segregation tools provides new insights to further the understanding of the complex geography of segregation during working hours. While the high isolation values obtained by our analysis confirm known patterns of residential segregation documented for Santiago [41–43], our approach proposes means to look beyond static representations of such phenomena by inspecting the dynamic processes of social interactions at the destination of commuting journeys. This will be increasingly possible as the availability of detailed spatio-temporal mobile phone data increases. Approaches as the one proposed here will hence allow for a much better understanding of the structure of the city when compared with analyses from origin-destination or other conventional surveys [11,14,44,45]. In particular, the communities found here confirm sociological descriptions that roughly divide Santiago into a rich part in the foothills to the east, and a less affluent zone to the west and south [41,42]. For instance, community C closely matches the so-called ‘high rent cone of Santiago’ described elsewhere [46]. Such cone-shaped community C may be thought of as the footprint of the upper-class zones described for Latin American cities in such literature [47], where it has been proposed to emerge from the historical movement of the elites out of downtown areas (usually central) towards arbitrary radial directions in the periphery. While we have no means to evaluate the effect of the built environment upon such segregation patterns, we do provide means to quantitatively evaluate how commuting dynamics may explain the socioeconomic composition of community C where the vast majority of SEL S1 and S2 is concentrated (figure 1*c*). Communities A, D and E instead, locate to the west, composing the less affluent social fabric of the city (see also their respective socioeconomic composition in figure 1*c*). Community F contains the most densely populated boroughs (*comunas*) of La Florida and Puente Alto, seen by some scholars as sub-central outgrowths [48] where most of the middle-class families live. Finally, community B represents the downtown Santiago, and it has a more diverse socioeconomic composition, which is in correspondence with the fact that people stemming from all communities converge within it (figure 3), making community B the only one in which urbanites show no statistical segregation at their workplaces, as opposed to the other five communities where urbanites are likely to find co-workers belonging to their same community.

While economic factors have largely been recognized as key processes shaping social interactions, we highlight that the large segregation found among peripheral communities are not simply given by their geographical context or individuals’ decisions to minimize costs, by moving to the nearest places, for example. Our null model explicitly considers costs associated with the commuting of urbanites by drawing random distances from the empirical distribution of each community. Moreover, because the directionality (i.e. *theta*) of each community is also used, the centripetal effect, that is, the observed tendency of people to work in the centre of the city, is also considered in our null model. Thus, finding higher values of RII with respect to SII is indicative of a segregation (or isolation) pattern between communities that is not merely explained by the geographical context of individuals, but most likely driven by a social tendency to move towards specific locations where the encounter probability between members of your own community is enhanced when compared with a randomized scenario.

These highly segregated communities raise new concerns when considering the central role of skill complementarity upon production factors thought to lower segregation in more economically productive areas [2,49,50]. Our results emphasize the importance of assessing segregation not only from a spatial and static point of view, but also using temporal assessments of segregation, such as proposed by Silm & Ahas [2], where temporal changes of ethnic exposure levels are assessed from mobile phone data across the day. It is important to note, however, that our approach is not able to explain the causes of the spatial segregation of communities. In fact, post-hoc analyses using complementary sources of data (e.g. census data) will be instrumental to such aim. For instance, productivity has been syndicated a major driver to attract workers, portraying commercial or industrial zones as more integrated, and less segregated, compared to residential areas. Also, polycentric or subcentre patterns in cities (e.g. the southern zone in the case of Santiago [48]) can lead to generate centripetal effects not only towards the centre of the city, but also to the commercial core of that community. In this sense, a closer look at the spatial distribution of productivity at the neighbourhood level could certainly shed further insights on causal relationships of the segregation patterns described here. While such endeavours are beyond the scope of our method, it will most likely disentangle potential effects ascribed to road infrastructure on the centripetal pattern observed in our model city, Santiago.

Finally, it is important to notice that interaction probabilities represented by the isolation index employed here assume that each person within a Voronoi cell will interact with all other individuals in the cell with the same probability, which may not be correct for all cases (e.g. people from office buildings interact much more with their workers than with the flower seller outside their building), hence finer data could reveal that isolation between communities is even higher than obtained here.

## 5. Conclusion

In this work, we show the benefit of developing new approaches to understand dynamic processes of social segregation. We show how the exposure dimension of segregation can be successfully studied from the increasingly available cellphone registries by combining network analysis with segregation indexes. Although we focus on the exposure levels at workplaces, this framework is easily extensible to the whole activity space of individuals if specific values of social interaction between groups may be found for each hour of the day (e.g. leisure activities after work may foster social interaction compared with residential patterns). Because it only relies on cellphone data, our methodology stands as an effective means to compare a wide range of cities of different sizes and characteristics, irrespective of the particular differences emerging from the close-up analysis of socioeconomic data coming from different countries and/or methodologies. Nonetheless, the specific case of Santiago developed here corroborates how our analysis provides useful information that may be combined with other sources of data, such as socioeconomic level or other census data, which may be used by urban planners, politicians and social scientists in general to get further insights into the structural and functional patterns of cities.

## Supplementary Material

Communities by day; Home-work trajectories for each mobile phone user

## Data accessibility

Dataset containing users’ home and work locations as well as community affiliation of each tower are available from Dryad Digital Repository: http://dx.doi.org/10.5061/dryad.9p4r16m [51].
