# Supplementary material for: The time geography of segregation during working hours
Source: R Soc Open Sci. 2018 Oct 3;5(10):180749. doi: 10.1098/rsos.180749 (PMC6227938; doi:10.1098/rsos.180749)
Supplement: Communities by day; Home-work trajectories for each mobile phone user [file rsos180749supp1.pdf]

Supplementary Material:
The time geography of segregation during working hours

Teodoro Dannemann

teodoro.dan@gmail.com

Boris Sotomayor-Gómez

bsotomayor92@gmail.com

Horacio Samaniego*

horacio@ecoinformatica.cl

Ecoinformática Lab
Universidad Austral de Chile
Campus Isla Teja, Valdivia, Chile

1 Sensibility analysis of community detection algorithm

The XDR dataset consists of four weeks (from Monday to Friday) distributed along four months: March, May, October and November. We assessed the changes of communities detection as a function of the working day chosen by comparing the results to the "aggregated network", that is the full dataset . We generated five home-work networks using the procedure outlined in Section 2. The first network was created aggregating data of all Mondays (i.e. March 16, May 11, September 3, and November 23), the second network was produced with all available Thursdays, and so on. We then applied the community detection algorithm explained also in Section 2. Results show that, six communities were always found in Santiago, irrespective of the day chosen to perform the analysis, and with a large match rate when compared to the aggregated network (see Figure S1). To quantify the correlation among resulting communities, each of the six communities were labeled in the aggregated network. Then, quantified for each detected community, the number of the nodes remaining in the same community when compared to the aggregated case. The resulting correlations are shown in Table S1. Despite of community B, which changes from the downtown zone in the aggregated case into a broader area in the case of Tuesday to Friday, the communities retained their ascription to the same, and well defined, zones (see Figure S1).

	**Monday**	**Tuesday**	**Wednesday**	**Thursday**	**Friday**
**A**	78.81 %	61.02 %	75.42 %	63.56 %	66.10 %
**B**	71.30 %	74.07 %	69.44 %	79.63 %	83.33 %
**C**	83.55 %	69.30 %	76.54 %	67.32 %	74.12 %
**D**	73.76 %	92.57 %	91.58 %	80.20 %	80.69 %
**E**	93.46 %	94.39 %	95.33 %	88.79 %	88.79 %
**F**	84.21 %	85.53 %	79.61 %	88.82 %	93.42 %

Table S1: Percentage of nodes retaining their same community ascription, compared to the aggregated network.

*Corresponding author

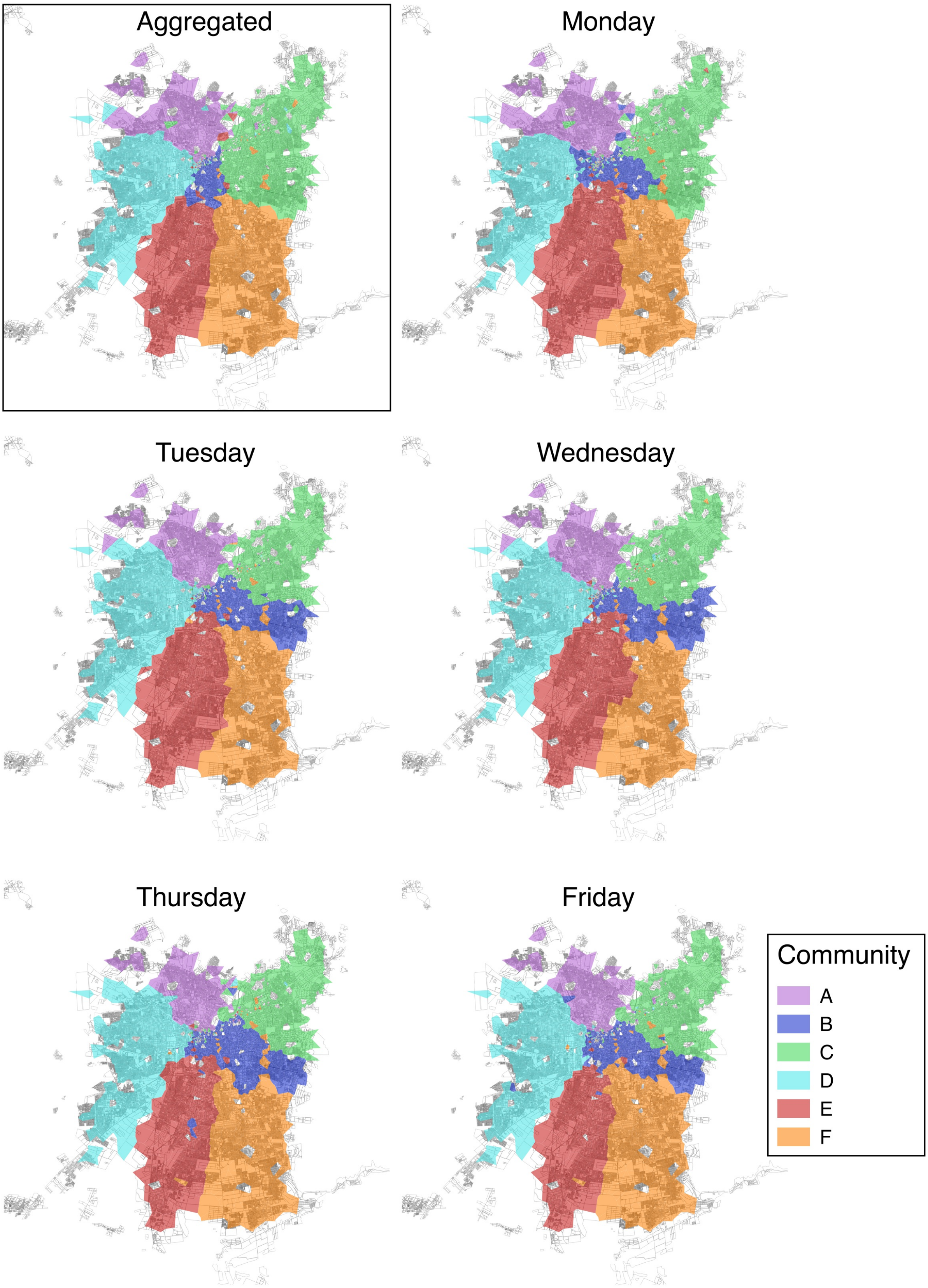

Figure S1: Comparing the communities obtained for each weekday network. The upper left map (outlined) is the aggregated network, i.e., the network created by taking all the data.

2 Random walk simulations

We also developed an algorithm to create new simulated workplaces for each user in our dataset. The algorithm generates random coordinates from the empirical distance and movement angle distribution for the community that the user belongs to. A pseudo-code description is provided:

Algorithm 1 Random walk simulations algorithm

procedure RANDOMWALKS(*communities*,*dataset*)
  for each community *comm* **in** *communities* **do**
    $dist_d$, $dist_\theta \leftarrow$ get distance and angle movement probability distribution for community *comm*
  for each user u **in** *dataset* **do**
    $X_h, Y_h \leftarrow$ get homework coordinates of user u
    $d, \theta \leftarrow$ assign a random distance and angle of movement to user u, which are taken from probability distributions $dist_d(u)$ and $dist_\theta(u)$, respectively.
    $X'_w \leftarrow X_h + d \cdot \cos(\theta)$
    $Y'_w \leftarrow Y_h + d \cdot \sin(\theta)$
    if (X'_w, Y'_w) lies outside the urban boundary **then goto** line 6
    else assign the closest tower to point (X'_w, Y'_w) as the new workplace of user u

3 Socioeconomic level classification from census data

We used the classification of socioeconomic level (SEL) proposed by ADIMARK (2009). Family households are classified in one of five categories: $ABC1$, $C2$, $C3$, D, and E, which in our work, relabeled as $S1$, $S2$, $S3$, $S4$, $S5$ and $S6$ for simplicity. $ABC1$ are the most affluent families. This labeling follows from the criteria described in Table S2. Hence, two dimensions drive the SEL classification: educational level and ownership of material goods.

**Educational level**	**0**	**1**	**2**	**3**	**4**	**5**	**6**	**7**	**8**	**9**	**10**
No studies	E	E	-	E	E	D	-	D	D	C3	C3
Elementary (incomplete)	E	E	-	E	E	D	-	D	C3	C3	C3
Elementary (complete)	E	E	D	-	D	D	D	C3	C3	C3	C3
Secondary (incomplete)	D	D	D	D	D	D	D	C3	C3	C3	C2
Secondary (complete)	D	D	D	D	C3	C3	C3	C3	C2	C2	C2
Technical (incomplete)	C3	C3	C3	C3	C3	C2	C2	C2	C2	C2	ABC1
Technical incomplete or Higher Education incomplete	C3	C3	C3	C3	C3	C2	C2	C2	C2	ABC1	ABC1
Higher (complete)	C3	C3	C3	C3	C3	C2	C2	C2	ABC1	ABC1	ABC1

Table S2: ADIMARK Socioeconomic classification matrix. Columns are number of household goods (Shower + Color TV + Refrigerator + Washing machine + Hot water system + Microwave oven + Satellite/Cable TV + PC + Internet + Car).

Both dimensions are directly extracted from census data. Each census block is then represented by the most frequent SEL in that block (i.e. geographic unit).

4 Calculation of socioeconomic level for each community

Each census block k may be represented by a vector $\vec{v}_k$ of five components, one per each socioeconomic level (SEL), and it will have a value of one for the corresponding SEL, and zeros in all the other four places (each census block is represented only by one SEL). For example, if the census block k belongs to the $S1$ category, then $\vec{v}_k = [1,0,0,0,0]$. Given a Voronoi cell j, with area A_j, we may obtain its socioeconomic composition $\vec{v}_j$ as:

$$\vec{v}_j = \sum_k \frac{A_{kj}}{A_j} \vec{v}_k, \tag{1}$$

where $A_{kj} = A_j \cap A_k$, the areal intersection. This assures that area is taken into account, and Voronoi cell composition is a mere aggregation of census blocks weighed by their area contribution. In Figure S2 we show an example, where we have five blocks (red rectangles) contributing to a Voronoi cell (in blue). Red filled areas correspond to the intersection areas A_{kj}. In this case, $S1$ and $S2$ are the main SEL. It is easy to see that the sum of the elements of $\vec{v}_j$ is less or

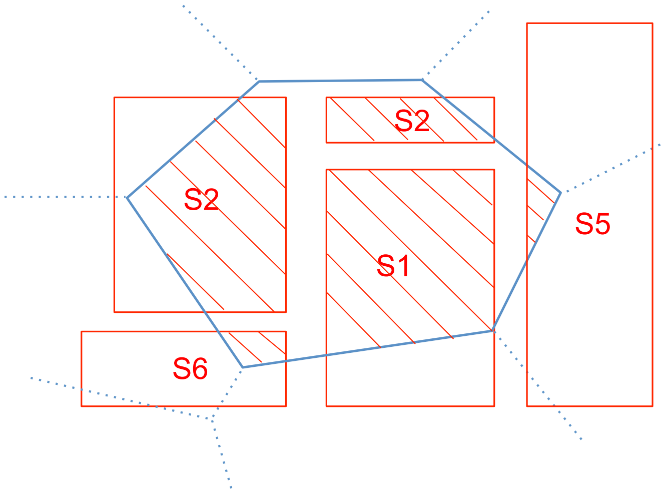

Figure S2: Example of a Voronoi cell superposed with census blocks.

equal to one. However, if we want to interpret the elements of $\vec{v}_j$ as a proportion (or percentage) of a certain SEL, there is one more issue to deal with. As it can be seen in figure S1, the equality $\sum_k A_{kj} = A_j$ is not always fulfilled, because there are gaps between census blocks (red rectangles), so that the entire area of the Voronoi cell is not filled. These gaps usually correspond to streets, parks, or other non residential areas. Nonetheless, one can easily correct this by redefining each vector $\vec{v}_j$:

$$\vec{v}_j' = \frac{\vec{v}_j}{\vec{v}_j \cdot \vec{1}} \tag{2}$$

i.e., to do this correction and interpret area coverage as a percentage we may divide $\vec{v}_j$ by the sum of its elements to adjust the resulting sum to one.

Finally, we may obtain the SEL composition vector for an entire community i as follows:

$$\vec{v}_i = \frac{\sum_{j \in i} \vec{v}_j' A_j}{\sum_{j \in i} A_j} \tag{3}$$

where the sum is taken over all the Voronoi cells j belonging to community i.

References

[1] Adimark GFK. Mapa socioeconómico de Chile; 2009.
